# ‘Responsive Dialogues’, an engagement framework for antimicrobial resistance: a cross-country qualitative evaluation in Thailand and Malawi

**DOI:** 10.1080/16549716.2026.2676473

**Published:** 2026-06-05

**Authors:** Bhensri Naemiratch, Anne Osterrieder, Mackwellings Phiri, Phaik Yeong Cheah, John Mankhomwa, Tassawan Poomchaichote, Raymond Pongolani, Supanat Ruangkajorn, Thomasena O’byrne, Kanpong Boonthaworn, Deborah Nyirenda, Eleanor MacPherson

**Affiliations:** a Mahidol Oxford Tropical Medicine Research Unit, Faculty of Tropical Medicine, Mahidol University, Bangkok, Thailand; b Centre for Tropical Medicine & Global Health, Nuffield Department of Medicine, University of Oxford, Oxford, UK; c Malawi Liverpool Wellcome Research Programme, Blantyre, Malawi; d Liverpool School of Tropical Medicine, Liverpool, UK

**Keywords:** Participatory dialogues, LMICs, community engagement, policy, global health

## Abstract

Antimicrobial resistance (AMR) is a global health crisis requiring multisectoral responses that extend beyond biomedical interventions. Participatory approaches can ensure that policies and practices reflect community realities, yet evidence from low- and middle-income countries (LMICs) remains limited. To examine how the Responsive Dialogues (RD) framework was implemented in Malawi and Thailand, and to identify lessons on adapting participatory methods for AMR engagement, managing power dynamics, overcoming structural barriers, and supporting policy uptake. This cross-country evaluation applied the RD toolkit developed by the Wellcome Trust, which included three phases: groundwork, community conversations, and post-conversation sharing. Both countries followed this framework but adapted it to their social and political contexts. Data included field observations, facilitator reflections, participant feedback, and analysis of policy outcomes. Four themes emerged across both sites. First, adaptation was engagement process which shaped by trust, flexibility, and cultural sensitivity. Second, entrenched hierarchies influenced participation; strategies such as stakeholder briefings, ground rules, and rotating leadership roles helped mitigate imbalances, while in Malawi, village leaders’ authority supported antibiotic regulation. Third, although Malawi’s RD recommendations informed its revised National Action Plan on AMR, Thailand’s community generated ideas were less visible in the national strategy, underscoring persistent gaps between local insights and high-level policy. Finally, reflective practice strengthened iterative learning and inclusivity. Overall, RD offers a promising model for facilitating participation in AMR mitigation plan. However, the differences in policy uptake in both countries highlight the limits of participatory approaches when not aligned with policy scale and resourcing.

## Paper Context

- **Main findings:** Responsive Dialogues enabled inclusive dialogue across community and policy stakeholders in Malawi and Thailand, demonstrating how relational adaptation, power-aware facilitation, and reflective learning are essential for effective participatory AMR engagement
- **Added knowledge:** This study offers new empirical evidence from LMIC settings on how participatory methods can surface community-generated solutions, navigate hierarchical constraints, and support pathways for policy influence in AMR policies.
- **Global health impact for policy and action:** Strengthening institutional support and resourcing for participatory engagement can help embed community insights into AMR policy design and implementation, promoting more equitable, sustainable, and context-responsive strategies worldwide.

## Background

Antimicrobial resistance (AMR) is increasingly recognised as one of the most complex global health threats, with profound implications for human, animal, and environmental health [1,2]. It is driven by a wide range of interconnected factors, including inappropriate antimicrobial use in human and veterinary medicine, agricultural practices, weak regulatory systems, and broader socio-economic determinants [3,4]. A recent modelling study estimated that cumulatively from 2025 to 2050, over 39 million deaths globally could be attributable to antibiotic resistance, and 169 million deaths associated with it [5,6]. The highest burden is predicted to fall on low- and middle-income countries (LMICs), where health systems are often under-resourced [4,6].

Tackling AMR requires multisectoral ‘One Health’ approaches that move beyond biomedical interventions to address its social, political, and economic dimensions [7–10]. Community engagement is increasingly recognized as essential for ensuring interventions are trusted, contextually relevant and responsive to lived realities [11–15]. Community perspectives help identify feasible solutions, including for marginalized groups, and strengthen public legitimacy [16–18]. Integrating community voices with stakeholder collaboration across health and agriculture can enhance policy uptake and sustainable AMR governance [19,20].

The *Responsive Dialogues on Drug-Resistant Infections* (RD) framework, developed by the Wellcome Trust, supports structured and inclusive conversations on AMR by bringing together policymakers, scientists, community members, and practitioners to deliberate on evidence and co-create feasible solutions [21]. Grounded in participatory research and co-creation principles, RD emphasises inclusivity, reflexivity, and systems thinking to ensure responses are rooted in lived experience rather than solely biomedical expertise [11,22,23]. The approach strengthens the AMR evidence base by capturing public knowledge, attitudes, and priorities, while identifying practical actions communities consider achievable. RD, therefore, offers a mechanism for bridging the persistent gap between community perspectives and policy processes, aligning with growing calls for AMR governance that foregrounds social and behavioural drivers [24].

The toolkit is organised into three phases: groundwork (contextual analysis and stakeholder engagement), community conversations (participatory workshops to surface challenges and co-create solutions), and post-conversation impact (sharing outputs with policymakers and supporting translation into action). These phases facilitate attention to structural barriers such as healthcare access and agricultural practices while promoting community-led, evidence-informed responses.

## Methods

### Cross-country adaptation

The Thailand and Malawi teams were awarded funding from Wellcome for piloting the RD toolkit because they had key research sites with existing Wellcome-funded research and public engagement, faced significant public health challenges like AMR in resource-limited settings, and, therefore, offered strong platforms to test innovative community engagement to bridge policy and implementation gaps in tackling complex issues. The project implementation was led independently by country teams, but both teams collaborated throughout the study period as of a shared learning process guided Wellcome Trust. This included regular cross-country meetings to compare operational pathways, discuss adaptations to the Responsive Dialogues toolkit, and reflect on emerging challenges and enabling conditions.

In this study, Responsive Dialogues (RD) is defined as a participatory engagement approach and implementation framework designed to facilitate structured dialogue between communities, policymakers, and technical experts to co-create context-specific AMR solutions. Implementation of the RD framework required extensive contextualisation in both Malawi and Thailand, reflecting distinct social, political, and institutional environments. This includes relational adaptation which we define as the process of adjusting engagement practices through trust-building, negotiation of roles, and responsiveness to social and institutional dynamics, rather than through technical modification alone. While both countries followed the same three-phase RD framework, the operational pathways diverged significantly, as each team tailored engagement strategies to their respective cultural and governance contexts. Table 1 provides an overview of the key adaptations in each country.

**Table 1.** Key adaptations in the implementation of the ‘Responsive Dialogues’ framework in Malawi and Thailand.

Method	Malawi	Thailand
Overarching aim	To co-create solutions and policy asks with key stakeholders in both policy and with community members To inform the revision of the Malawi National Action Plan on AMR with co-created, context-specific insights [25]	To improve understanding and engagement with AMR among adult Thai communities To inform the national action plan on AMR policy to include co-created, context-specific solutions To improve understanding about the practicalities of implementing RD toolkit in Thailand
Team background	Core team: 6 (including principal and co-investigators, project coordinator, facilitators, evaluator) External partners: Head of National AMR Unit	Core team: 6 (including principal and co-investigators, project coordinator, facilitators, evaluator) External co-applicants/partners: 2 (AMR researchers/policy experts) External facilitator team: 3 (lead facilitator and 2 co-facilitators)
Mapping the AMR ecosystem	Stakeholder mapping conducted at project outset to identify policy, health, and community actors	Stakeholder mapping conducted at outset and updated during implementation to help identify regional participants and national policy key stakeholders
Target audiences	National policy makers including AMR policy makers within Ministry of Health, Ministry of Agriculture – Animal Health District level policy makers Health workers (including community prescribers) Community groups including farmers, prescribers and male care givers	Government staff working on AMR Public health personnel Local area administration Civil society (e.g. non-governmental organisations) Adult community members (incl. agriculture, business, vulnerable groups) Communication and media actors
Monitoring and evaluation	Monitoring: Participant feedback surveys Pre- and post-workshop surveys Reflective staff meetings after each session	Monitoring: Participant and staff feedback and reflections collected directly after the events (e.g. through surveys) Regular staff meetings to monitor progress and quality of the project Keeping in touch with participants between events (e.g. ‘LINE’ group)
Monitoring and evaluation	Evaluation [25]: Iterative feedback information session 15 semi-structured interviews with workshop participants from 10 focus group discussions, held at two time points to capture changes	Evaluation: Continuous feedback loop [26] Participant short feedback form at the end of each conversation 4 focus group discussions 12 individual interviews [27]
Community conversation	Planning conversations with key stakeholders by conducting in-person Community conversations events, 3–4-day workshops All events held in Blantyre	Planning conversations with key AMR actors (two in-person, one virtual) Community conversations: two national, virtual events (adult and youth); four regional, in-person events (Northeastern, Northern, Southern and Central regions)

This study adopts a comparative qualitative design across two LMIC settings. The cases were selected pragmatically as part of a multi-country initiative, enabling comparison of how RD operated under differing policy and governance conditions. The aim was not formal hypothesis testing, but to generate transferable implementation insights. Participants were purposively selected to represent key stakeholder groups involved in AMR, including policymakers, health professionals, and community members. Sampling aimed to capture diverse perspectives across sectors and levels of decision-making. Data sources included observations, facilitator reflections, participant feedback, interviews, and focus groups. Triangulation was achieved by comparing findings across these sources to identify consistent themes and discrepancies. Table 1 provides an overview of the key adaptations in each country.

## Results

This paper presents findings from the between-site comparisons in these countries and are presented around four thematic areas: 1) Contextual adaptation of the RD toolkit; 2) Managing power dynamics; 3) Overcoming societal, political and economic barriers; and 4) Enabling reflective practice (Table 2).

**Table 2.** Themes and subthemes identified.

Theme	Subthemes
1. Contextual adaptation of the ‘Responsive Dialogues’ toolkit	a. Stakeholder mapping and engagement
	b. Tailoring tools and facilitation methods
	c. Adapting meeting formats
2. Managing power dynamics	a. Challenges
	b. Strategies to support participation
3. Socio-political and economic constraints	a. Tensions between bottom-up aspirations and top-down realities
	b. Policy uptake of co-created solutions
4. Reflective practice and learning	a. Iterative learning process
	b. Culture of continuous learning

### Theme 1: contextual adaption of the ‘Responsive Dialogues’ toolkit

In this theme, we discuss the ways in which we adapted to the RD framework to the two very different contexts we were working in.

#### Stakeholder mapping and engagement

In Malawi, stakeholder identification and engagement were central to contextualising the RD framework. The team worked with the AMR Coordinating Unit at the Ministry of Health and the African Institute for Development Policy (AFIDEP) to align with existing initiatives and build a stakeholder network. Using snowball sampling, they expanded participation to include policymakers, scientists, non-governmental organisations (e.g. World Health Organisation), private sector actors, media, and visual artists involved in health communication.

In Thailand, the RD project coincided with the development of the Second Thailand National Strategic Plan on AMR (NSP-AMR 2023–2027) [28]. With support from a project team member with strong AMR networks and external facilitators (Civicnet), ‘Planning Conversations’ were convened that included members of the NSP-AMR subcommittee, government officers, healthcare providers, and AMR researchers (e.g. Division of Medical Disease Control, Chulalongkorn University) [27]. These discussions helped map the Thai AMR ecosystem, identify priority stakeholders and issues for the community conversations, align the project with the NSP-AMR, and review past AMR campaigns to inform co-created communication solutions.

#### Tailoring tools and facilitation methods

Communicating the complexity of AMR to diverse participants posed significant linguistic and conceptual challenges in both countries. In Malawi, the term ‘antimicrobial resistance’ had no direct translation in local languages, requiring creative facilitation to avoid technical jargon. The ‘drug bag’ exercise [29] presenting assorted antibiotic packages familiar to participants proved particularly effective for sparking discussion and surfacing local experiences of antibiotic access and use. The exercise encouraged farmers, prescribers, and caregivers to share knowledge without fear of judgement, while AMR experts on-site clarified misconceptions in real time.

Similarly, in Thailand, facilitators modified techniques to match linguistic nuances and cultural norms. AMR was often confused with ‘anti-inflammatory’ medicine [27] leading to conceptual misunderstandings. To address this, the team produced short educational videos featuring a senior AMR researcher, paired with simple graphics and Thai subtitles to demystify AMR science. The videos, which explained the science of AMR and its impact in Thailand, were shown at the start of the community conversation to help set a shared understanding. Mindfulness practices and local storytelling were also incorporated into workshop schedules to create comfortable, non-hierarchical learning spaces. These adaptations ensured accessibility and reinforced inclusivity, aligning with previous research showing that participatory communication improves engagement and comprehension in AMR interventions [23,30].

#### Adapting meeting formats

Both countries faced disruptions due to COVID-19, which demanded flexible redesign of engagement formats. The planned national stakeholder workshop in Malawi was replaced by one-on-one interviews, held either in person around Lilongwe and Blantyre, or by telephone. Blantyre was chosen for its accessibility and proximity to the research institute. This decentralized approach allowed flexibility and iterative learning, with in-person meetings following strict safety protocols. Scheduling was adjusted to participant availability, and facilitators used exploratory questions to draw out existing knowledge and experiences of antibiotics and AMR, rather than beginning with instructional content.

In Thailand, the project team adopted a two-pronged approach with both virtual and in-person events to overcome logistical challenges. When new government restrictions were imposed after the initial planning event, the team shifted subsequent national youth and adult conversations online, using the digital collaboration platform ‘Padlet’ [31] to map existing AMR engagement activities. The Thai team also conducted national adult and youth conversations online [27]. For these, the RD guidance was adapted to develop new activities, grounded in the team’s previous experience in facilitating virtual meetings with a Bangkok community advisory board during COVID-19 restrictions [32]. The online events were attended by participants who might otherwise have been excluded due to health conditions, competing commitments, or travel barriers.

### Theme 2: managing power dynamics

A key feature of the RDs is how they bring together the perspectives of community members and other stakeholders. However, managing complex power dynamics to ensure inclusivity and diversity of voices posed a challenge in both settings. Here we describe how social hierarchies shaped by age, professional status, gender, or community roles affected group dynamics in both countries, and how we addressed these challenges.

#### Challenges of hierarchy and participation

Power relations strongly influenced participation in both Malawi and Thailand. In Malawi, social hierarchies position village heads as community authorities and men as primary decision-makers, while scientists and policymakers are highly respected due to their roles and expertise. Communities are often accustomed to being research subjects rather than active contributors, limiting confidence to question experts or share unfamiliar opinions [33]. To address this, high-power stakeholders were briefed prior to events and encouraged to create space for community perspectives. Initial engagement was cautious as participants negotiated expectations, but confidence increased over time as familiarity with the process grew, illustrating the importance of continuity and trust-building in shifting hierarchical norms.

In Thailand, respect for seniority and credentialed expertise presented visible challenges. Senior professionals at times dominated discussions or used technical language, which risked excluding non-experts. On occasion, solutions proposed by community members were discounted or sessions shifted into Q&A formats led by health professionals, limiting co-creation. Participants reported feeling that the space sometimes prioritised expert perspectives rather than shared learning.

Overall, cultural norms around authority and expertise required proactive facilitation strategies to support equitable dialogue. Even with deliberate mitigation, hierarchies remained influential, underscoring the need for long-term capacity-building to embed more participatory ways of working.

#### Strategies to support participation

Both teams employed multiple strategies to mitigate the influence of hierarchy and enhance equitable participation. Facilitators were trained in culturally responsive moderation, including rotating group leadership roles and intentionally structuring seating or break-out groups to distribute influence.

In Malawi, pre-event briefings with village heads, policymakers, and experts clarified that the conversations prioritised community knowledge. Ground rules on respectful listening were co-developed with participants, and facilitators actively encouraged quieter individuals while limiting dominant contributions. Power was also strategically leveraged: village leaders were recognised as important actors for enforcing community by-laws on antibiotic sales. Local drama and roleplay helped participants and stakeholders visualise the feasibility of proposed solutions – such as safe disposal of livestock – and elicited empathy by illustrating the lived realities driving antimicrobial use.

In Thailand, similar strategies were implemented. Stakeholders were briefed prior to events, and ground rules emphasised shared ownership. Break-out groups were designed to minimise hierarchical influence by separating experts from lay participants and mixing senior and junior colleagues across groups. Neutral facilitators ensured balanced discussion and prevented sessions from reverting to expert-led Q&A formats.

Despite these measures, power dynamics continued to surface, highlighting the ongoing need for intentional facilitation and iterative adaptation to uphold inclusivity throughout the process.

### Theme 3: socio-political and economic constraints

While the RD process successfully facilitated deep engagement and co-creation of context-specific ideas, the translation of these ideas into tangible and actionable solutions was frequently constrained by broader social, economic, and political barriers. In this theme, we discuss broader issues that need to be considered in any future AMR engagement activities.

#### Tensions between bottom-up aspirations and top-down realities

Across both Malawi and Thailand, a shared outcome of the RD process was the strong emphasis on fostering community-led AMR initiatives. The timing of the projects coinciding with national reviews of AMR Action Plans created a strategic opportunity to introduce public perspectives into what are typically highly centralised policy processes. In many LMICs, including Malawi and Thailand, health policies have historically been designed through top-down approaches, with decision-making concentrated among ministries and technical actors and limited avenues for meaningful public participation. Communities are often positioned as end-users rather than co-producers of policy.

In both countries, however, AMR policy units, the AMR Coordinating Unit in Malawi and the FDA/MOPH structures in Thailand, expressed a desire to strengthen bottom-up engagement, recognising the value of community perspectives for enhancing policy relevance and ownership. Despite this openness, the RD process revealed persistent tensions between hierarchical governance cultures and the emerging preference for participatory, community-driven approaches. While participants voiced a clear desire to shape AMR interventions, many continued to rely on government officials, health workers, and traditional leaders to legitimise or lead actions. This reflected long-standing expectations that authority lies with formal powerholders and highlighted the difficulty of operationalising community-led initiatives in settings shaped by deference and centralised leadership norms.

#### Policy uptake of co-creation solutions

Both sites also encountered challenges when attempting to translate co-created ideas into implementable solutions, particularly where participants expected material support or additional resources. Turning community insights into policy-ready proposals required navigating institutional constraints, budget limitations, and differing expectations around roles and responsibilities.

In Malawi, direct involvement of policymakers proved crucial. Members of the AMR Coordinating Unit attended RD sessions, listened to community perspectives, and relayed these insights within government structures. This helped ensure that several RD recommendations, such as improving antibiotic sale regulation, were incorporated into the revised National Action Plan on AMR. A final national stakeholder workshop brought together policymakers, scientists, media actors, and additional stakeholders identified through the process, reinforcing collaboration and maintaining momentum beyond the project period.

In Thailand, the team and participants hoped to pilot selected co-created solutions to generate evidence for policymakers, but resource constraints prevented this. Although the team presented findings at the *Third National Forum on AMR (2022)* and contributed to drafting Strategy 5 of the NSP-AMR 2023–2027 [27], the community-level recommendations were not incorporated into the final policy. This reflected the inherent gap between high-level strategic planning [28] and the granular, locally focused nature of RD-generated solutions.

The analysis suggests that RD influenced outcomes through engagement process and institutional mechanisms such as trust building, legitimacy, and policy alignment whose effects were mediated by broader health system governance, resource availability, and timing within national AMR policy processes.

### Theme 4: reflective practice and learning

This theme explores how reflective practice and supporting ongoing learning improved the quality of engagement, and enabling adaptation of the RDs in real time. Across both country settings, teams embedded structured and informal reflection mechanisms throughout the RD process, contributing to its responsiveness, relevance, and long-term value.

#### Iterative learning process

Reflective practice was integrated throughout the RD process via structured debriefs, team meetings, and facilitator reflection logs after each community conversation. These mechanisms allowed teams to identify issues related to group dynamics, language clarity, participant engagement, and to rapidly adapt facilitation tools and approaches. Such iterative learning was crucial for navigating the unpredictability of multi-stakeholder dialogue in resource-constrained settings.

In Thailand, monitoring and evaluation were embedded from the outset [26]. Participant feedback through surveys and discussions informed real-time adjustments; for example, shortening regional dialogues from three to two days to improve accessibility and attendance. Digital communication platforms enabled continued interaction between events, ensuring a sustained feedback loop and maintaining engagement over the project period.

#### Culture of continuous learning

A culture of continuous learning helped both teams to remain responsive to unexpected developments, whether logistical, interpersonal, or contextual, and ensured that the RD process did not follow a rigid script but evolved alongside the experiences of participants. Teams also used feedback mechanisms such as participant surveys, group discussions, and digital communication platforms to gather insights between events. This allowed for a continuous feedback loop and helped maintain engagement beyond the physical dialogue spaces. Reflections were not limited to process efficiency, but extended to questions of equity, power, and participation, such as how to include less vocal participants, when to adjust language for clarity, or how to support trust-building across groups. Learning was continuously integrated into the planning and delivery of activities.

The learning from our two pilot projects informed the implementation of RD in Zambia led by ICARS (International Centre for Antimicrobial Resistance Solutions) and subsequently Vietnam and South Africa. The original Wellcome Trust RD toolkit has since been updated by ICARS by adding training modules and guidelines [21], and the RD framework has been embedded in AMR engagement activities in Thailand and other countries.

## Discussion

This cross-countries, cross-sectional qualitative evaluation examined the implementation of RD in Malawi and Thailand, demonstrating how participatory approaches can strengthen AMR engagement and link community perspectives with policy processes in LMICs. Across both setting, adaptation emerged as both technical and engagement process, shaped by trust-building, responsiveness, and sensitivity to local context. In Malawi shifted to one-on-one interviews during COVID-19, while Thailand adopted a hybrid online in-person model to sustain participation. These iterative adjustments reflect wider evidence that engagement must evolve with context to remain inclusive [23,29,30]. However, adaptation alone could not overcome broader structural constraints.

While RD enabled meaningful engagement in both settings, their effects differed markedly. In Malawi, early and sustained involvement of policymakers, alignment with a National Action Plan review and strong institutional anchoring, supported partial policy uptake. In Thailand, despite rich community dialogues and co-created solutions, the approach did not translate into comparable policy influence. Differences in policy uptake between Malawi and Thailand are interpreted as context-specific associations rather than causal effects of RD, shaped by institutional alignment, policy timing, and resource availability. Community-level proposals were difficult to integrate into a high-level national strategy, and limited post-dialogue resources constrained follow-up and piloting. These findings indicate that RD is most effective when participatory processes are embedded within policy cycles, supported by institutional ownership, and resourced beyond dialogue events. Where these conditions are absent, RD may strengthen understanding and engagement, but is unlikely to generate systemic change. Participatory engagement alone is insufficient; effectiveness depends on governance conditions and institutional embedding.

Power dynamics operated at both individual and system levels. At individual interaction level, participation was shaped by social norms and positional authority. Cultural deference to chiefs and experts in Malawi, and respect for seniority in Thailand, risked silencing community perspectives [34,35]. Structured facilitation strategies including stakeholder briefings, ground rules, and rotating roles – helped mitigate dominance. At the same time, existing authority structures could support change: village leaders in Malawi enabled discussions on antibiotic access [36].

At the system level, policy uptake varied across sites, depending on institutional alignment, resourcing and policy architecture. While Malawi integrated RD recommendations into the revised National Action Plan on AMR, Thailand, findings informed national discussions, yet locally specific solutions did not fully translate into the final NSP-AMR due to its macro-strategic focus [3,37]. Table 3 provides a comparative synthesis of implementation conditions and observed outcomes across Malawi and Thailand.

**Table 3.** Summary of Responsive Dialogues implementation: outcomes and conditions shaping impact across study sites.

Domain	Malawi	Thailand
Policy context	Active NAP-AMR revision	High-level NSP-AMR development
Policymaker engagement	Early and continuous involvement	Episodic engagement
Community co-creation	Context-specific solutions developed	Context-specific solution developed
Observed policy engagement	RD findings discussed and reflected in the policy process	RD findings presented in national forums and consultations
Post-dialogue follow-up	Stakeholder dissemination and continued engagement	Limited follow-up dur to resource constraints
Implementation constraints	Limited resource for follow-up activities	Misalignment with policy scale; limited resources

Socio-economic constraints also limited feasibility. Farmers’ reliance on antibiotics and expectations of material support highlights the need to embed participatory AMR efforts within broader health, agricultural, and livelihood systems [12].

Finally, integrating reflective practice through debriefs and feedback loops helped maintain responsiveness and strengthened inclusivity throughout implementation [38].

Based on these findings, Table 4 outlines priority recommendations for implementing RD in future AMR engagement initiatives.

**Table 4.** Priority recommendations for implementing Responsive Dialogues on AMR.

Align dialogues with policy cycles	Engagement should be timed to coincide with active policy review processes and include early, sustained involvement of decision-makers to enable translation of community insights into formal policy.
Strengthen facilitation for inclusive participation	Facilitators should be trained to manage social hierarchies, support marginalised voices, and strategically engage authority figures, recognising power as both a constraint and a potential resource.
Support post-dialogue pathways	Participatory processes require dedicated resources beyond dialogue events, including support for piloting co-created solutions and mechanisms linking community outputs to institutional action.
Embed reflective learning throughout implementation	Structured reflection, feedback loops, and adaptive learning should be built into programme design to ensure responsiveness, inclusivity, and relevance across diverse contexts.

## Limitations

This project has several important limitations. First, as a cross-sectional qualitative evaluation, it cannot establish causal relationships between RD and changes in behaviour, policy, or AMR outcomes. The findings, therefore, identify plausible mechanisms and enabling conditions rather than causal or long-term impacts. Second, much of the evidence on practices and perceived impacts is based on self-reported data from participants and facilitators, which may be subject to social desirability and recall bias, particularly in group settings involving authority figures. Third, the study did not include longitudinal follow-up or independent observation of practice change, limiting assessment of durability and real-world implementation.

While the RD framework includes post-conversation implementation and piloting of co-created solutions, in both countries the post-conversation phase focused primarily on policy-level engagement rather than community-level pilots. Resource and time constraints limited the feasibility of implementation within the study period. Nevertheless, co-created solutions and policy asks were synthesised and shared with national stakeholders, contributing to the revision of Malawi’s NAP-AMR and informing Thailand’s NSP-AMR process. Future longitudinal and mixed-methods research is needed to assess causal pathways, behavioural outcomes, and system-level impacts of participatory AMR initiatives over time. Finally, in both countries, the project primarily focused on antibiotic resistance, not the whole spectrum. We did not discuss antiviral, antifungal, or anti-parasitic resistance.

## Conclusions

This cross-country evaluation shows that Responsive Dialogues (RD) can meaningfully strengthen AMR engagement by creating inclusive spaces for communities, policymakers, and technical experts to deliberate and co-create solutions. In Malawi, strong institutional alignment and sustained policy maker involvement enabled community insights to inform national AMR policy. In Thailand, RD primarily enhanced dialogue and awareness, with limited policy uptake.

Effective implementation required careful adaptation to local context, where trust-building, flexibility, and cultural sensitivity were as critical as technical design. Participatory approaches alone were insufficient: translation into action depended on policy timing, institutional ownership, and post-dialogue resources. RD should, therefore, be understood as a facilitating mechanism whose impact is contingent on broader governance conditions rather than a standalone solution. To maximise impact, participatory AMR approaches require long-term support, institutional buy-in, and explicit mechanisms that bridge community priorities with system-level action.

## Data Availability

Data underlying the paper may be requested from the Mahidol-Oxford Tropical Medicine Data Access Committee (email: datasharing@tropmedres.ac).
